# RLRL Therapeutic Feasibility and Potential Mechanism on Myopia

**DOI:** 10.3390/ijms27010428

**Published:** 2025-12-31

**Authors:** Yu-Jiao Chen, In-Chul Jeon, Seung-Sik Cho, Dae-Hun Park

**Affiliations:** 1 Department of Optometry, Dongshin University, Naju 58245, Jeonnam, Republic of Korea; yujiaoch@gmail.com (Y.-J.C.); icjeon@dsu.ac.kr (I.-C.J.); 2 Biomedicine, Health and Life Convergence Sciences, BK21 Four, College of Pharmacy, Mokpo National University, Muan 58554, Jeonnam, Republic of Korea; 3 College of Korean Medicine, Dongshin University, Naju 58245, Jeonnam, Republic of Korea

**Keywords:** myopia, repeated low-level red light (RLRL), therapeutic mechanism

## Abstract

Myopia is a major global public health concern, with a particularly high and increasing prevalence in East Asia. Although significant progress has been made in regard to developing strategies to slow the progression of myopia, the precise biological mechanisms underlying the onset and progression of myopia remain unclear. Repeated low-level red light (RLRL) therapy, a novel non-invasive photobiomodulation (PBM) technique, has demonstrated promising efficacy for controlling axial elongation and refractive error progression. This review first outlines the clinical definition, epidemiology, and global health impact of myopia, followed by the etiology-based pathogenesis and corresponding intervention strategies. Special attention has been given to emerging mechanistic evidence supporting RLRL, particularly its role in activating mitochondrial cytochrome c oxidase (CCO), enhancing retinal metabolism, influencing choroidal changes in blood perfusion and thickness, and in scleral remodeling. Finally, the feasibility and potential mechanism of the RLRL therapy for slowing myopia progression have been discussed from the perspective of safety.

## 1. Introduction

Myopia is defined as the focus of parallel light rays in front of the retina with relaxed accommodation [1,2]. Although myopia was first recognized as a correctable refractive error years ago, it is increasingly considered a progressive and vision-threatening disease. This change in concept is not only due to the rapid increase in the prevalence of myopia worldwide, but also because it is associated with irreversible structural complications of the eye, including myopic maculopathy, posterior staphyloma, glaucoma, and retinal detachment [3]. The subjective symptoms of myopia are that distant objects are not clear, but nearby objects are relatively clear. After using the eyes for a long time, it is easy to feel visual fatigue, such as eye soreness. Individuals with myopia tend to squint while looking at distant objects. In this situation, less light entering the pupil causes the image enhancement from aberrations to decrease with small pupil diameters [4], and squinting increases the depth of field [5]. The objective symptoms of myopia identified by ophthalmologists or optometrists indicate that the unaided vision of patients with myopia fails to satisfy these criteria for normal vision, prior to optical correction. In addition, the length of the eye axis has gradually become an important reference indicator in the assessment of myopia [6]. Most ocular complications caused by myopia are associated with high myopia. Patients with high myopia usually exhibit excessive eye-axis elongation. High myopia is no longer regarded as a simple refractive error, but as a complex, multifactorial eye disease that may even cause irreversible vision loss. It is also closely related to genetic susceptibility, environmental factors, and abnormal neurosensory signal pathways [7,8,9].

RLRL therapy is a photobiomodulation (PBM)-based therapy and a non-drug intervention for myopia control [10]. The wavelength range of light used for PBM therapy, including far-red (FR) and near-infrared (NIR) light, spans 630–1000 nm [11]. Ruby (694 nm) and HeNe lasers (633 nm) were used for PBM in the early stages, but no actual laser was required later because the same effect could be achieved with incoherent light-emitting diodes (LEDs) [12]. For myopia prevention and treatment, the RLRL wavelength range is 650 ± 10 nm (illuminance approximately 1600 Lux) using a laser or LEDs [13]. The threat of myopia is not limited to the individual level. Relevant surveys have shown that myopia affects visual health worldwide, and there are clear differences across countries and regions.

In 2024, Liang et al. reported the trend and forecast of myopia prevalence among global adolescents and children between 2000 and 2050; they estimated that the global myopia prevalence would increase from 22.9% of the population to 49.8%, with the growth rate almost doubling, and the number of myopic patients would increase from approximately 1.4 billion to 4.758 billion. One of the most important prospects is that the proportion of individuals with high myopia will increase from 163 million (approximately 2.7% of the world’s population) to 938 million (approximately 9.8%) during the same period. This means that the total prevalence of myopia is expected to double, whereas the prevalence of high myopia is expected to increase fivefold [14]. East Asia has the highest prevalence and most serious burden of myopia. Among people in the same age group, the prevalence of myopia among East Asians is more than twice that among Caucasians [15]. For example, compared with other regions, the prevalence of myopia in Seoul, Singapore, Hong Kong, and Taiwan has remained high for a long time. Among high school graduates in these regions, the incidence of myopia can exceed 80%, and the prevalence of high myopia is as high as approximately 20%, far exceeding the global average [8].

Although there were regional differences in myopia prevalence, areas with the highest concentrations of myopia and high myopia were also the most densely populated. This indicates that the number of people with myopia was high, and as the proportion of myopia increased, the likelihood of developing high myopia also increased. The chain reaction that follows is related to eye complications and irreversible vision damage, and myopia problems in this large group of people largely represent the global burden of myopia. According to forecasts, by 2050, nearly 10% of the world’s population will have high myopia, which will also cause direct or indirect losses of all types. In addition to vision damage, the overall burden caused by myopia includes (1) medical costs, such as eye examination fees, the cost of refractive correction glasses, and the cost of treating myopia-related complications [16,17,18] and (2) loss of productivity due to visual impairment, including unemployment and missed work. In 2017, Naidoo et al. reported that to address visual impairment caused by refractive error, sufficient ophthalmic manpower and ophthalmic and optometry examination facilities were needed, and an investment of US $20 billion was expected over 5 years. Current estimates also found that the productivity loss caused by myopia is far more than 10 times the investment required, and the productivity loss is expected to exceed US $200 billion each year (even as the most conservative estimate, the return on investment was still found to be 250%) [19]. Therefore, if the global burden of myopia prevention and control is effectively addressed, considerable economic benefits can be generated. There are many misunderstandings regarding the pathogenesis of myopia, and many studies on the causes of myopia have been conducted [20]. Several causes of myopia have been suggested, including the combined effects of genetic susceptibility [21], environmental factors [7,8], accommodation [22,23,24,25], peripheral defocus [26,27,28], contrast sensitivity [29,30], and axial elongation caused by choroidal [13,19,31] or scleral changes [8,32,33]. Interventions related to myopia control include behavioral strategies, pharmacological treatments, optical solutions, and RLRL treatment using photobiomodulation technology.

Implementing behavioral strategies usually requires altering the routine lifestyle; it is difficult for students under academic pressure to follow day after day. Although optical and pharmaceutical interventions for myopia have been widely adopted, these methods mainly target upstream mechanisms of the visual pathway. For instance, spectacles or contact lenses improve the clinical symptoms of myopia rather than resolve the downstream biological processes underlying scleral remodeling and abnormal eye growth [1,34,35]. This recognition has stimulated interest in exploring novel therapeutic strategies that can intervene at the cellular metabolism and intercellular signaling levels, including photobiomodulation therapy [36].

Until now, improving myopia has been difficult, but many trials are underway to establish the appropriate method to correct it. Although RLRL therapy was suggested as one of them, the therapeutic mechanism of that on myopia was not clear. In this study, we presented the feasibility and potential mechanism of the RLRL therapy for slowing myopia progression.

## 2. Myopia

### 2.1. Classification of Myopia

#### 2.1.1. Classification by Anatomical Features

Axial myopia is a myopic refractive state that can be attributed to excessive elongation of the eye axis. The other is a myopic refractive state that occurs with changes in the image formation structure or position of the eye, called refractive myopia. Refractive myopia includes index, curvature, and anterior chamber myopia [2,37].

#### 2.1.2. Classification According to Degree of Refraction

Although there are slight differences in the standards for myopia degree between many documents, more than half of the 145 myopia-related documents have defined myopia and high myopia as −0.50 diopter(D) or less and −5.00 D or less, respectively [38].

#### 2.1.3. Classification Divided by Physio-Pathological Factor

Physiological myopia is a type of common myopia that is not associated with eye disease; it progresses steadily and is considered nonpathological myopia [2,39]. Pathological myopia is defined as degenerative myopia with major changes occurring in the posterior segment of the eye globe [2,40]. There is currently no precise definition of pathological myopia, and it is uncertain whether high myopia will develop into pathological myopia or if they are two diseases with different underlying causes [41].

### 2.2. Factors Associated with the Development of Myopia

#### 2.2.1. Genetic Susceptibility Background

Myopia is a multifactorial disease influenced by both environmental and genetic factors [8]. Large-scale genome-wide association studies (GWAS) and meta-analyses have identified hundreds of susceptibility loci associated with refractive errors and myopia. For example, this meta-analysis has increased the number of independent signals identified from 37 to 161 and revealed a shared genetic background for myopia susceptibility among different ethnic groups [42]. The gap junction protein delta 2 (GJD2) gene plays a key role in retinal neurotransmission and in eye growth regulation [21]. Another important gene is ras protein-specific guanine nucleotide-releasing factor 1 (RASGRF1), which is involved in retinal neurotransmission, function, and development. RASGRF1 is strongly associated with myopia risk in European and Asian populations [21].

The prevalence of myopia has surged rapidly within a brief timeframe, particularly in East Asian nations, which has been insufficient for the gene pool to change due to this epidemic trend, and there is not much solid evidence linking myopia to genetic causes.

#### 2.2.2. Outdoor Time

Outdoor activities are an important and effective intervention strategy to prevent the onset of myopia in children and adolescents [8]. Seven cross-sectional meta-analyses highlighted that increasing outdoor activity time by one hour per week can reduce the risk of myopia by 2% [43]. In 2015, a three-year school cluster randomized controlled trial in China revealed that adding 40 min of outdoor activities significantly reduced the incidence in 6-year-old children for the next three years [44]. The cumulative incidence of myopia was 30.4% and 39.5% in the intervention and control groups, respectively. After three years, the spherical equivalent refraction (SER) of the children in the intervention group was significantly lower than that in the control group. The mean change in the intervention group was −1.42 D and in the control group was −1.59 D, with a difference of 0.17 D (95% CI, 0.01 D to 0.33 D, *p* = 0.04).

#### 2.2.3. Environmental Scenes

Differences in environment and lifestyle will also have a great impact on the prevalence of myopia.

In addition to time spent outdoors, different light intensity levels may affect myopia. A study evaluating the relationship between dim light exposure and myopia analyzed the differences in myopic and non-myopic children exposed to four different levels of light after wearing light sensors. The results showed that myopic children were exposed to significantly less dim light for night vision on weekends than non-myopic children (*p* = 0.024) and also received significantly less outdoor bright light (*p* < 0.001). These findings suggest that dark light exposure is associated with the regulation of retinal signals and the development of the eye. The development of myopia is influenced not only by bright light exposure but also by low-light stimulation [45].

A previous bidirectional Mendelian randomization study, using large-scale genomic data from 67,798 individuals, explored the causal relationship between additional years of education and myopia. The results showed that for each additional year of education, myopia increased by an average of approximately −0.27 D (95% CI −0.37 to −0.17; *p* = 4 × 10^−8^). In contrast, the change in myopia per diopter of refractive error had little effect with additional years of education [46].

#### 2.2.4. Accommodation

The focusing strength of the eyes is 0 D if we watch an object from five meters away, whereas looking at nearby objects generates various degrees of accommodation [2]. Over the past 18 years (2006–2024), Schachar developed and continuously refined an accommodation-related biomechanical model, asserting that changes in the ciliary muscle’s adjustment tension affect the abnormal deformation of the lens, which indirectly causes abnormal accommodation and promotes the progression of myopia [24,47]. Although this differs from the classical theory of Helmholtz [48], this perspective provides a plausible explanation for the pathogenesis of accommodation-associated myopia. However, there are currently many different accommodation-related hypotheses to explain the progression of myopia, such as the lag of accommodation hypothesis [49,50,51], tonic accommodation theory [52,53], and the microfluctuation abnormality hypothesis [54].

#### 2.2.5. Peripheral Hyperopic Defocus

Emmetropia means that, in healthy eyes, the image is correctly focused on the retina under the appropriate refractive status. Myopic individuals obtain blurred views as the images are defocused in front of the retina [5]. In 2007, Smith et al. conducted a study on rhesus monkeys and focused on the importance of the peripheral retina in affecting the refractive state [55]. One study revealed that controlling the defocus of the peripheral retina can change the growth and refractive state of the eye [26]. Peripheral hyperopic defocus leads to axial myopia, while peripheral myopic defocus leads to axial hyperopia [28].

#### 2.2.6. Contrast-Theory-Related On–Off Pathway Stimulation

Viewing high-contrast targets is more likely to continuously stimulate the retinal OFF pathway [56] and trigger a stronger contrast adaptation effect [57]. Strong stimulation of the OFF pathway leads to choroidal thinning [58], and the thinner the choroid, the higher the degree of myopia [59,60,61,62]. By contrast, lower contrast sensitivity inhibits the development of myopia [63]. Moreover, relevant genetic studies have shown that children with mutations in the myopia-related gene locus, ${MYP}_{1}$ would have abnormally increased retinal contrast signals, which may be the cause of high myopia. Thus, it can be inferred that abnormally high retinal contrast (possibly caused by genetic or environmental factors) is a signal that drives eye growth [29,30].

#### 2.2.7. Axial Elongation (Sclera and Choroid)

Axial elongation is a crucial pathogenic factor that contributes to the onset or progression of myopia, and can be driven by an imbalance in complex physiological regulatory mechanisms. In regard to the progression of myopia, myopiagenic visual signals may diminish the production of scleral collagen and elevate the synthesis of enzymes that degrade the scleral extracellular matrix (ECM), resulting in remodeling of the scleral ECM [7]. This may result in substantial thinning and enhanced elasticity of the sclera, leading to excessive elongation of the ocular axis [8].

However, the sclera lacks nerve innervation, necessitating the choroid to serve as an intermediary for the transmission of retinal signals that regulate eyeball growth. First, the choroid may secrete growth factors to promote scleral growth. Second, choroidal thickness may influence the transmission function, which potentially acts as a barrier. Third, the change in choroidal volume may be influenced by internal vascular filling and matrix water content. During this period, the choroid provides mechanical pressure to the sclera. The sclera experiences mechanical pressure from the choroid, and the interplay of several biochemical signals, including collagen degradation, can accelerate alterations in the scleral structure, thereby influencing the growth rate of the ocular axis [31]. Choroidal thickening may push the retina forward, thus reducing the axial length of the eye [64].

### 2.3. Interventions for Myopia Control

#### 2.3.1. Progressive Addition Lenses

As early as 1999, Leung et al. found that progressive addition lenses (PALs) could slow the progression of myopia and speculated that the interaction between PALs and the accommodation system may slow the progression of myopia [65]. Subsequently, a 3-year study in the United States of diverse ethnic backgrounds evaluated the effects of PALs when compared to conventional single-vision lenses (SVLs) on myopia progression in adolescents. It was found that using PALs slowed myopia progression in children by a small but statistically significant amount when compared to SVLs [66]. However, the research group of Berntsen found that the core mechanism of PALs in slowing myopia is that it can change the peripheral defocus state of the eyeball, especially producing myopic defocus on the upper retina, thereby providing a signal to stop excessive eye growth [67,68,69].

#### 2.3.2. Highly Aspherical Lenslet Target and Defocus-Incorporated Multiple Segment Lenses

Highly aspherical lenslet target (HALT) and defocus-incorporated multiple segment (DIMS) lenses for functional frame glasses, designed to induce peripheral myopic defocus for decreasing myopia progression, have shown promising efficacy. In a two-year trial [54], the DIMS lens reduced myopia progression by 52% and axial elongation by 62%, whereas the HALT lens, which has 11 rings of aspherical microlenses, achieved reductions of 67% and 60% in high-compliance wearers [70,71]. Dual-focus design soft contact lenses (DFCL) employ a bifocal design featuring a +2.00 D defocus in the outer ring, which has been shown to reduce myopia progression by 59% and axial elongation by 52% on average over a three-year period [72].

#### 2.3.3. Diffusion Optics Technology Lenses

Diffusion optics technology (DOT) lenses are covered in microscopic diffusers throughout the lens. Each microscopic diffuser is approximately 0.14 mm in diameter and 0.2 mm in height. Its irregular shape features a flat top and relatively steep sidewalls. Only approximately 5 mm of the central clear aperture, aligned with the pupil, is retained. These lenses prevent myopia by reducing the contrast signal of adjacent cones through the microscopic diffusers, thereby inhibiting axial eye growth.

DOT lenses grounded in the retinal contrast hypothesis can diminish refractive progression by 74% after a year, showing the potential for fine-tuning central-peripheral visual stimulation [73].

#### 2.3.4. Orthokeratology Lenses

Orthokeratology lenses (OK lens) modify the central curvature of the cornea during nocturnal wear, generating peripheral myopic defocus with an efficacy rate of approximately 40–60% [74,75,76]. A five-year follow-up research indicated that the average axial length increase in the OK lens group was 0.99 mm, markedly lower than that of the conventional lens group at 1.41 mm [77].

#### 2.3.5. Atropine

Currently, atropine eye drops are the only extensively used medications for myopia management in pediatric patients. Prior research has shown that 1% atropine can reduce the progression of myopia by over 70% after two years. However, side effects, including eye photophobia and accommodation problems, restrict its regular application [78]. In contrast, a low concentration of atropine (0.01%) has fewer side effects while retaining its efficacy and has become a mainstream choice. On average, 0.01% atropine can slow refractive progression and axial lengthening by approximately 59% within two years [79,80].

#### 2.3.6. Repeated Low-Level Right Light (RLRL) Therapy

Repeated low-level right light (RLRL) therapy is a novel myopia therapy with a positive effect on axial length control in children with myopia. According to previous findings, after 1 year of irradiation with red light of a wavelength of 650 ± 10 nm, the proportion of axial length (AL) significantly shortened (>0.05 mm) and reached 21.6% [34]. Another study found an effect of RLRL therapy in adult patients with myopia [13]. Adult patients aged 18–35 years were selected for the RLRL method to intervene in the progression of myopia. More than half of the subjects in the RLRL group had an axial length decrease of more than 0.05 mm, while the axial length of the single-vision lens wearers in the control group did not change significantly.

Although the aforementioned factors are the focus of current research on the causes of myopia and have gradually been confirmed to be closely related to myopia, these causes are still insufficient to explain the complex pathogenesis and individual differences in myopia. An increasing number of studies have pointed out that the formation of myopia is not driven by a single factor but involves multiple mechanisms. Currently, research on the underlying mechanisms of myopia remains limited, especially for identifying the most effective and accurate intervention.

The occurrence and development of myopia are closely related to the midstream (retinal nutritional metabolism and transduction) and downstream (choroid and scleral) of the visual pathway, and the new RLRL treatment method achieves its therapeutic effect by regulating this key link. Therefore, the new therapy of RLRL in photobiomodulation (PBM) therapy studied in this article is an attempt to explore the precise target of myopia intervention mechanism.

## 3. RLRL Therapy

### 3.1. Evidence from Non-Clinical Studies

For the evaluation of the myopia-controlling effect of RLRL application, many studies have been conducted using several species, such as chicks [81], guinea pigs [82], tree shrew [83], and non-human primates, like rhesus monkeys [84,85]. The neonates of most animals are hyperopic [86]. However, the results on the relationship between light wavelength and emmetropization in neonates vary and are contradictory. In infant rhesus monkeys, red light (630 nm wavelength) prevents hyperopia defocus by shortening the vitreous chambers and choroidal thickening to inhibit axial elongation of the eye [85]. In a tree shrew study, red light (626 ± 10 nm wavelength, steady and flickering) may induce hyperopia, and steady blue light (464 ± 10 nm wavelength) exposure did not improve hyperopia, but flickering blue light radiation significantly induced myopic refraction and elongation of the vitreous chamber [83]. Conversely, chicks [81] raised under red light conditions gradually develop myopia, whereas those raised under blue light conditions gradually develop hyperopia, and flashing red light induces myopia gene expression in guinea pigs [87].

When red light therapy is used as an intervention for myopia control in different animal models, we have observed varying results, with some even exhibiting opposing trends. This phenomenon suggests that the same intervention method may not respond identically across different biological systems. Differences in ocular anatomy, refractive systems, and the rate and stage of ocular development across species may influence the sensitivity of interventions. Furthermore, subtle differences in light source wavelength and illuminance within each experimental design may also amplify interspecies discrepancies in results. For instance, in guinea pigs exposed to a 750 nm, 800 lux red flash with a 2 s on/2 s off (≈0.25 Hz) pattern, axial length elongation and a decrease in SER of −11.29 D were observed, leading to significant myopia [87]. In the experiment on tree shrews, a steady-state LED red light of 626 ± 10 nm and 527 lux was used for overhead illumination, and it was found that the tree shrews maintained significant hyperopia [82]. In the experiment on infant rhesus monkeys, a narrow-band long-wave LED red light of 630 nm and 274 ± 64 lux was used for illumination, and it was found that the narrow-band red light promoted hyperopia and significantly slowed down myopia and suppressed axial length growth [85].

### 3.2. Potential Mechanism of RLRL Therapy

**Figure 1:**
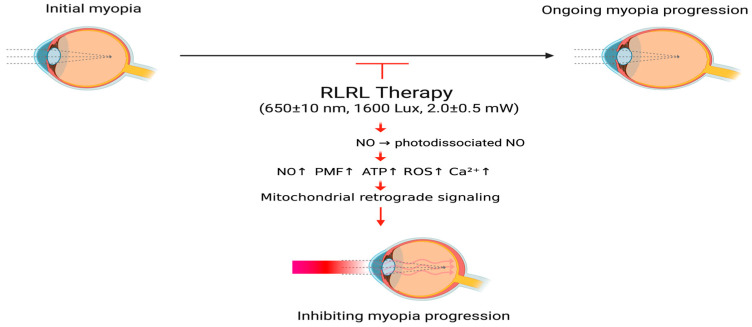
The proposed mechanism of RLRL therapy for myopia. RLRL therapy activates CCO activity through PBM, causing photodissociation of NO, triggering mitochondrial retrograde signals, promoting ATP production and improving energy supply and cell metabolism of ocular tissues, causing choroidal vasodilation and increased blood flow, and regulating scleral remodeling; thereby delaying axial growth and slowing the progression of myopia. RLRL, repeated low-level right light; NO, nitric oxide; PMF, proton motive force; ATP, adenosine triphosphate; ROS, reactive oxygen species; ${Ca}^{2+}$, calcium ions. Created in BioRender. Chen, Y. (2025) https://BioRender.com/e1hxynj.

The basic theoretical hypothesis of RLRL therapy for controlling myopia is that RLRL may have biological effects that delay the growth of the eye axis and the progression of myopic refractive diopter by (1) accelerating retinal cell metabolism [74,86,88], (2) modulating physio-anatomical choroidal changes such as thickening and increasing blood flow [32,87], and (3) mediating scleral remodeling [1,10,13,88]. Figure 1 shows the mechanism of action of RLRL therapy in myopia.

The above-mentioned basic theoretical hypotheses are all centered on changes in the light signal transmission process under the action of photobiomodulation (PBM) in eye-related cells. The mechanism that triggers the core “mitochondrial retrograde signal” is the cytochrome c oxidase (CCO) on the inner membrane of the mitochondria [89,90]. CCO is abundantly distributed throughout the visual system, such as in the retina and the central visual pathway, and serves as a metabolic marker of neuronal activity [91].

For nerve cells in the retina and brain, this oxidase is also a key mitochondrial enzyme in cell bioenergetics, and CCO is also an important structure of the mitochondrial Electron Transport Chain (ETC) [92]. The ETC consists of five multiprotein complexes (Complexes I-V), each of which plays a unique role. Complex IV (cytochrome c oxidase, CCO) facilitates electron transfer to molecular oxygen [92,93]. The chemical permeation on the ETC can be understood as the electron transport chain transfers electrons and protons on the membrane, transferring electrons and protons from one side of the membrane to the other [93,94].

Complex IV (CCO) has been confirmed to be a photoreceptor for red light to near-infrared light (600–900 nm) and is the central medium for PBM treatment of myopia [36,89,95]. As the tail component of ETC, CCO plays a vital role in electron transfer and proton transport. In ECT, CCO is the only complex that directly interacts with oxygen molecules [88]. Subunit I of CCO is composed of three catalytic sites, namely heme a, heme $a_{3}$ and ${Cu}_{B}$ center. Oxygen reduction occurs here, which is also the main site for proton translocation coupling [96].

RLRL stimulates NO to photodissociate from CCO and amplifies its activity, which is considered the starting point for RLRL’s biological effects [11,36,97,98]. CCO serves as a target of RLRL as a biological photoreceptor and is involved in respiration [95,99]. RLRL with a wavelength of 650 ± 10 nm is in the visible red range and is considered to match the absorption spectrum of heme a_3_ and ${Cu}_{B}$ centers, delivering sufficient photon energy to facilitate the photodissociation of attached NO molecules [36]. This is the molecular foundation of photobiomodulation treatment (PBMT) [36,95,97].

Reactive oxygen species (ROS) levels are slightly increased due to the bio-optical stimulation of RLRL [92,100], and moderately increased ROS will not cause toxic reactions in cells but act as a second messenger to trigger related signal transduction in cells [97]. It is worth noting that excessive RLRL irradiation may lead to excessive production of ROS, which may have harmful effects on cells [10,88].

The concentration of ${Ca}^{2+}$ is influenced by multiple upstream factors, including NO, ROS, ATP, and membrane potential [101]. Alterations in ${Ca}^{2+}$ levels subsequently affect the activity or expression of various signaling mediators [10].

#### 3.2.1. Accelerating Retinal Cell Metabolism

RLRL irradiation induces the photodissociation of NO from CCO, permitting oxygen rebinding and finally accelerating mitochondrial respiration [36,95,97,98].

The activity of CCO was enhanced after RLRL irradiation, and electron transport chain (ETC) electron transfer in the mitochondria was accelerated [92]. The CCO acts as the terminal enzyme of the respiratory chain in the process of cellular metabolism [96]. The order of electron transfer on the ETC is Complex I/II → CoQ → Complex III → Cyt c → Complex IV (CCO), and CCO takes the electron from cytochrome c (Cyt c) through the ${Cu}_{A}$ center in subunit II, which is a double copper center [102]. Then it comes to heme a in subunit I. Finally, heme transfers the electrons to the binuclear center (BNC), which is the heme $a_{3}$/${Cu}_{B}$ center. This will be the catalytic site for oxygen reduction [103]. Under standard physiological settings, NO can transiently inhibit the $O_{2}$ reduction process by attaching to heme a_3_ or ${Cu}_{B}$, thereby occupying the binding site of oxygen molecules. This represents a reversible inhibitor, akin to a “brake mechanism” for modulating the strength of mitochondrial electron transport and respiratory rate [95]. At the BNC, CCO plays another major role in reducing oxygen molecules to water [103]. The redox reaction [104] generated by oxygen binding to BNC is as follows:

(1) $$4e^{-}+\;4H^{+}+\;O_{2}\to \;2H_{2}O\;(\Delta G<0)$$

Of the four “chemical protons” required for each O_2_ molecule consumed, two are delivered via the K-pathway, which are only involved in the early stages of the redox reaction, that is, the charge balance after the electrons enter the bimetallic center [103]. The K-pathway is then closed due to conformational changes, and CCO enters a highly exothermic phase, where the oxygen-oxygen bond in the oxygen molecule breaks at the BNC [105]. Each step of oxygen bond breaking here requires the uptake of chemical protons, while electrons flow to the BNC, and pumped protons move to the membrane gap via the D-pathway. Since the K-pathway is closed in the early stage of the redox reaction, the D-pathway is responsible for the supply of chemical protons and the transmembrane transport of pumped protons in the middle and late stages of the redox reaction [104,105].

Under RLRL corresponding to the acceleration of electron transfer, the proton pumping flux also increases. The proton motive force (PMF) established during this process increases [92]. PMF drives proton reflux and provides a better basis for ATP synthesis; thus, cyclic synthesis of ATP increases. The transmembrane transfer of protons utilizes the principle of chemical permeation coupling. The increase or decrease in electrons on the metal ions in the redox reaction, mainly caused by BNC, will affect local charge and conformation [94]. The transmembrane electrochemical potential difference generated by the transfer is called the proton motive force (PMF) [92] (PMF = ΔpH + ΔΨ). This PMF contains a pH gradient (ΔpH) and an electrical potential (ΔΨ), which means that the proton concentration outside the mitochondrial membrane is higher and H^+^ is more, and while the electrons are being transferred, H^+^ is pumped from the mitochondrial matrix to the membrane gap [92,100]. During this time, the H^+^ accumulation in the membrane gap carries a positive charge, which is relatively negatively charged compared to the inside of the membrane, thus forming a membrane potential difference [106]. The PMF formed by the combination of these two drives the proton reflux together, which also creates the basic conditions for the subsequent synthesis of ATP [92].

CCO itself does not synthesize ATP. As the terminal enzyme of mitochondrial ETC, CCO participates in redox reactions and pumps protons across the membrane, thereby using the energy of electron transfer and the ΔpH formed by proton transfer as the proton motive force. ATP synthase (Complex V) uses the electron motive force to drive protons back into the matrix, providing the necessary energy basis for the synthesis of ATP [94]. ATP synthase is also called F_0_-F_1_. It consists of two main components, of which $F_{0}$ is located in the inner membrane of the mitochondria and acts as a channel for protons, $F_{1}$ is the catalytic center protruding from the mitochondrial matrix and has a strong ability to hydrolyze ATP [94]. Protons flow back into the mitochondrial matrix through the $F_{0}$ domain of ATP synthase, and this process releases the energy stored in the PMF; ATP synthase uses this energy to cause conformational changes in the $F_{1}$ catalytic domain. Therefore, the conformational change of $F_{1}$ here is the key point of the ATP synthase catalytic cycle, which makes the cycle of ADP phosphorylation to ATP efficient [92,94].

**Figure 2:**
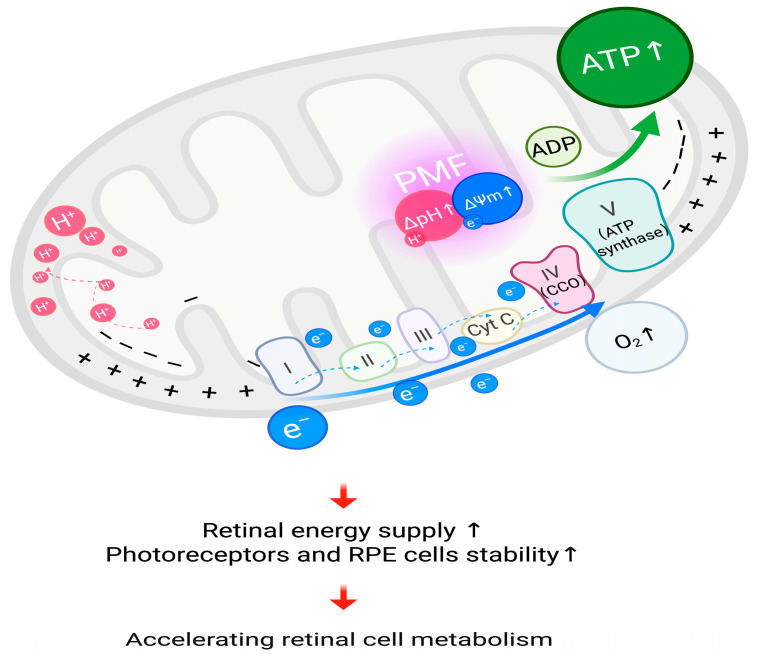
Accelerating retinal cell metabolism, RLRL irradiation is proposed to activate cytochrome c oxidase (CCO), leading to increased PMF, thus facilitating the cyclic synthesis of ATP, which leads to accelerated cell metabolism. ATP, adenosine triphosphate; $H^{+}$, proton; $e^{-}$, electron; ΔpH, pH gradient; ΔΨm, mitochondrial membrane potential; PMF, proton motive force; Cyt C, cytochrome c; $O_{2}$, oxygen; I, complex I of electron transport chain; II, complex II of electron transport chain; III, complex III of electron transport chain; IV (CCO), complex IV of electron transport chain (cytochrome c oxidase); V (ATP synthase), complex V of electron transport chain (adenosine triphosphate synthase); ADP, adenosine diphosphate; RPE, retinal pigment epithelium. Created in BioRender. Chen, Y. (2025), https://BioRender.com/pbwq3e6.

RLRL irradiation can improve mitochondrial metabolic function, increase ATP synthesis, and reduce oxidative stress, thereby enhancing the energy supply and stability of photoreceptors and retinal pigment epithelial (RPE) cells [11,107]. Figure 2 shows the process of accelerated retinal cell metabolism under RLRL treatment.

#### 3.2.2. Modulating Physio-Anatomical Choroidal Change, Such as Thickening and Increasing Blood Flow

Several past clinical studies have shown that RLRL increases the thickness of the subfoveal choroid by 10 µm in children [31,36,89]. This thickening was positively correlated with a shift toward emmetropia and reduction in axial lengthening, thus highlighting the key role of the choroid and sclera in RLRL-mediated myopia control [11,13,31].

Photodissociated NO enhances choroidal blood perfusion [75]. RLRL therapy increases NO concentration through photodissociation. NO acts as a vasodilator directly on the choroidal blood vessels [10,11]. The choroid is a vascular-rich tissue that contains a dense capillary layer, causing vasodilation and increased blood flow. The blood perfusion from the arteries of the arterial trunk in the Haller’s layer (large vessel layer) of the choroid, to the arterioles in the Sattler’s layer (medium vessel layer), and then to the innermost choriocapillaris capillary bed increases significantly. This leads to thickening of the choroidal tissue [31].

In addition, after RLRL irradiation, the pH in the cytoplasm increases, and the alkalinization effect activates the transient receptor potential vanilloid 4 (TRPV4) calcium channel, causing ${Ca}^{2+}$ influx [108]. The influx of ${Ca}^{2+}$ will activate calcium-sensitive nitric oxide synthases, such as endothelial nitric oxide synthase (eNOS) or neuronal nitric oxide synthase (nNOS), thereby enhancing the synthesis of NO [109]. NO stimulates soluble guanylate cyclase (sGC), leading to increased levels of cyclic guanosine monophosphate (cGMP), thereby activating protein kinase G (PKG) [11,95,109], which may lead to choroidal vasodilation and increased choroidal blood flow.

**Figure 3:**
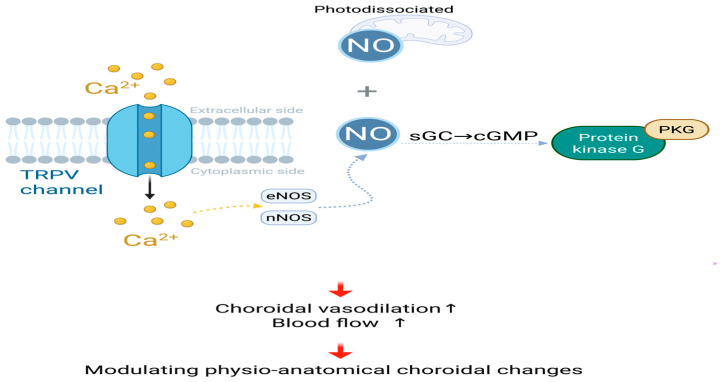
Modulating physio-anatomical choroidal changes, Photodissociated and calcium influx-induced NO jointly activate the sGC-cGMP-PKG signaling cascade, resulting in choroidal vasodilation and enhanced blood flow; NO, nitric oxide; ${Ca}^{2+}$, calcium ions; TRPV, transient receptor potential vanilloid; eNOS, endothelial nitric oxide synthase; nNOS, neuronal nitric oxide synthase; sGC, soluble guanylate cyclase; cGMP, cyclic guanosine monophosphate; PKG, cGMP-dependent protein kinase. Created in BioRender. Chen, Y. (2025), https://BioRender.com/p5soijs.

The growth rate of axial length slows down or shortens after using the RLRL treatment device, mainly because the expansion of choroidal blood vessels and the increase in blood volume will physically expand the choroidal tissue, thereby pushing the retina forward and shortening the effective axial length from the cornea to the retina [34,110]. In the research data of Zhou et al., after 9 months of RLRL treatment compared with the baseline, the sub-macular choroidal thickness (SFChT) thickened by 45.33 μm, the total choroidal area (TCA) increased by 0.05 mm^2^, and the cavity area (LA, representing the vascular component) increased by 0.05 mm^2^. However, the stromal area (interstitial tissue) was significantly reduced 7% approximately [111]. It is concluded that the thickening of the choroid is not an overall or uniform tissue expansion, but is mainly due to a significant increase in its vascular components, accompanied by a decrease in the matrix area. This non-uniform thickening method can cause the retina to move forward, ultimately slowing down the progression of myopia [13,111]. Figure 3 shows changes in physio-anatomical choroidal regulation under RLRL treatment.

#### 3.2.3. Mediating Scleral Remodeling

The RLRL treatment method can photodissociate inhibitory NO from CCO through the action of PBM. This relieves the inhibition of CCO and restores its redox activity [36,88]. Cellular respiration is then enhanced, and this sudden increase in respiration consumes the original oxygen in the tissue, thereby rapidly reducing the oxygen level [88,95]. Changes in cellular metabolism under hypoxia can induce the generation of ROS [112]. Under the stimulation of RLRL, CCO activity is enhanced, oxygen reduction is accelerated, thereby enhancing the overall electron flow rate and increasing the membrane potential (ΔΨm), thereby inducing reverse electron transport (RET) and leading to the generation of ROS [92]. During the RET process, the flavin mononucleotide (FMN) site of complex I, reduced flavin mononucleotide (${FMNH}_{2}$), easily leaks electrons to $O_{2}$ thereby forming superoxide anions ($O_{2^{•-}}$).

(2) $${FMNH}_{2}+O_{2}\to \mathrm{FMN}+O_{2^{•-}}+2H^{+}$$

The increase in ΔΨm increases the retention of semiquinone ($Q^{•-}$) at the ubiquinone oxidation ($Q_{o}$) site of complex III during the quinone cycle (Q cycle), and the unpaired electrons carried by $Q^{•-}$ are more likely to react with $O_{2}$ to generate $O_{2^{•-}}$ [100].

(3) $$Q^{•-}+O_{2}\to Q\;(\mathrm{ubiquinone})+O_{2^{•-}}$$

ROS, especially at appropriate concentrations, as important signaling molecules, can further stabilize HIF-1α [10,113]. Stabilization of hypoxia-inducible factor 1-alpha (HIF-1α) leads to the expression of vascular endothelial growth factor (VEGF) [114]. In animal experimental models, PBM (660 nm and 780 nm light) was observed to increase the expression of HIF-1α and VEGF in rat skin flaps, while reducing the activity of matrix metalloproteinase-2 (MMP-2), thereby promoting angiogenesis [64]. Additionally, the activation of HIF-1α may also be mediated by PBM acting on the signaling pathways of mitogen-activated protein kinase (MAPK) and phosphatidylinositol 3-kinase (PI3K)/Protein Kinase B (Akt) [87,95,115]. HIF-1α/VEGF pathway improves choroidal vasodilation and choroidal blood supply and then enhances oxygen supply to the sclera, which inhibits myopia-related scleral remodeling [33,88].

RLRL irradiation activates TGF-β/Smad signaling in scleral fibroblasts, leading to scleral ECM remodeling [116]. TGF-$\beta_{2}$ activates Smad2/3 through its receptor, increases the expression of collagen type I alpha 1 chain (COL1A1), improves scleral structural stability, and prevents excessive elongation of the eye axis [117]. TGF-β signaling may also induce the expression of α-smooth muscle actin (α-SMA), promote the transdifferentiation of fibroblasts into myofibroblasts, and lead to excessive fibrotic response. Photodissociated NO also improves local hypoxia, thereby inhibiting the abnormal activation of myofibroblasts and maintaining the dynamic balance of the scleral ECM [32,118].

**Figure 4:**
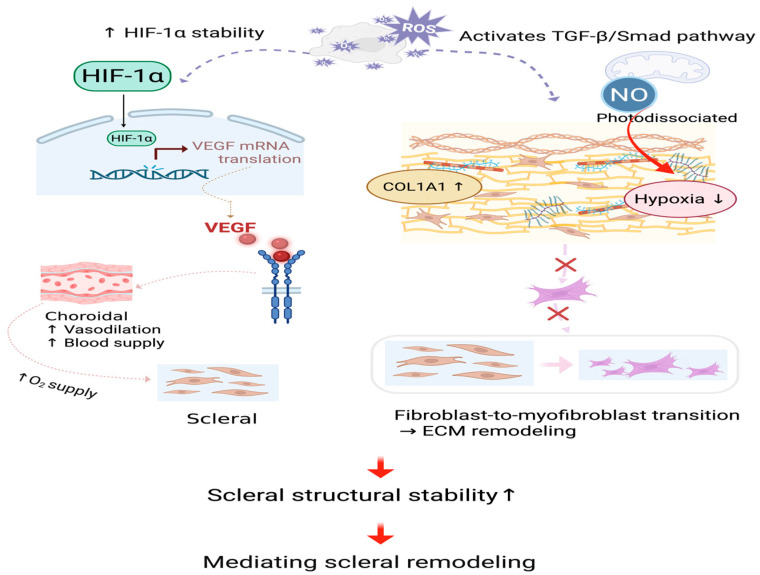
Mediating scleral remodeling. The HIF-1α/VEGF and TGF-β/Smad signaling cascades are activated by slightly increased ROS, which stabilizes the scleral structure. Dashed lines and cross marks denote schematic pathways that are attenuated or inhibited following RLRL treatment. NO, nitric oxide; ROS, reactive oxygen species; $O_{2}$, oxygen; HIF-1α, hypoxia-inducible factor 1-alpha; VEGF, vascular endothelial growth factor; mRNA, messenger ribonucleic acid; TGF-β, transforming growth factor-beta; Smad, small mothers against decapentaplegic; COL1A1, collagen type I alpha 1 chain; ECM, extracellular matrix. Created in BioRender. Chen, Y. (2025), https://BioRender.com/3cndwym.

Furthermore, the interplay between HIF-1α and TGF-β signaling exceeds a mere linear relationship [119]. HIF-1α signaling may interact with the TGF-β/Smad pathway in order to jointly regulate the stability of the sclera [120,121]. Figure 4 shows the process of scleral remodeling mediated by RLRL treatment.

## 4. Discussion

To date, RLRL therapy, a novel intervention for myopia progression control, is primarily indicated for adolescents and children. Existing randomized controlled/observational studies suggest significant efficacy in controlling myopic refraction and axial length growth, but evidence for long-term efficacy and safety, as well as important patient outcomes, remains limited. Regarding regulatory approval, RLRL therapy has been approved for myopia control in China and Australia [122]. Other countries/regions are starting the early clinical research or pilot phase, such as in June 2025, when researchers from Singapore published a study on the potential of PBM to slow the progression of myopia [123]. This article examines the potential mechanisms of RLRL within the framework of existing evidence and in combination with clinical efficacy.

Unlike existing optical and pharmacological interventions (e.g., OK lens, atropine, DIMS/HALT lenses, etc.) which mainly act through modify the visual input stimulus or visual signal transduction, RLRL therapy acts on myopia progression through alter retinal energy metabolism (mitochondrial oxygen status) and transmitters, falling under the gain modulation of the retinal transduction fuction; these effects may extend from the choroid to the downstream sclera, providing a new control path.

The potential mechanisms of RLRL therapy in myopia control remain under investigation. This article focuses on three possible pathways of action, but it should be noted that the underlying mechanisms extend far beyond these, requiring further investigation. For example, in the visual system, when symmetrical patterns are not salient, luminance polarity grouping can facilitate the visual system’s perception of symmetry [124]. RLRL, through light stimulation at a specific rhythm and frequency, may enhance visual symmetry perception, thereby enhancing the visual system’s processing of weak signals. Furthermore, recent studies on electroretinographic (ERG) patterns evoked by periodic stimulation in primates have found significant differences in the response to retinal function, with periodic stimulation being more strongly associated with activity in the posterior retinal layer and closely related to visual perception [125]. RLRL therapy, a periodic light therapy, and periodic ERG patterns can provide electrophysiological insights into the mechanisms of action of PBM. Therefore, future research is needed to further validate and expand upon these potential mechanisms to develop a more systematic and comprehensive understanding.

The mechanism of action of RLRL therapy warrants further attention and research. Its potential extends beyond myopia control. Glaucoma studies have shown that retinal ganglion cell damage reduces spatial frequency processing in the central visual field [126]. RLRL therapy may improve retinal cell energy metabolism and function, potentially helping maintain or restore spatial frequency processing in patients with early-stage glaucoma. Further exploration of the mechanisms of RLRL therapy promises to reveal further clinical applications in ophthalmology.

In clinical trials, RLRL therapy [127] has been usually performed with red light of a specific wavelength of 650 ± 10 nm, usually 5 days per week, twice a day for 3 min each time, with an interval of at least 4 h to intervene in the progression of myopia, and the subjects of the research are usually children and adolescents [34], but also adults [13]. The sample size typically ranged from dozens to hundreds of subjects [13,34,36,128]. Related experimental results have revealed that SER progression is significantly slower in the RLRL therapy group than in the control group [36,89]. AL grows more slowly or is shorter, and choroidal thickness increases compared to that before treatment [13,89].

According to previous findings, 12 months of RLRL treatment significantly slows AL growth and SER progression. The average AL growth in the RLRL group was 0.13 mm, while that in the SVL control group was 0.38 mm, and myopia progression was slowed by 69.4%. The SER progression in the RLRL group was −0.20 D, while that in the SVS control group was found to be −0.79 D, with a progression slowdown rate of 76.6%. Some children even experienced axial shortening: in the RLRL group, 39.8% of the participants had axial shortening ≥0.05 mm at 1 month, and this phenomenon was observed in 21.6% of the participants at 12 months. This study also found that the performed RLRL treatment could promote choroidal thickening: 1 month after treatment, the average choroidal thickness in the RLRL group increased by 16.1 μm [34]. Another study examined the effect of myopia prevention by using an RLRL treatment device in children with premyopia. At 12 months, in addition to the significant effects of AL and SER control, the effect of controlling the incidence of myopia was also obvious. The incidence of myopia in the RLRL intervention group was 40.8%, while that in the control group was 61.3%; the relative incidence of myopia was reduced by 33.4% [128].

Xiong et al. found that RLRL intervention combined with OK lens can achieve better therapeutic effects than a single method [129]. Based on the results of these clinical trials, no functional or structural damage to the photoreceptor layer was observed after RLRL treatment, and no persistent adverse visual experience was observed [34,35,128]. When compared to other myopia intervention methods, the RLRL treatment method is easier to use, especially for students with a heavy academic workload. The RLRL treatment device can be used to perform myopic interventions at home. The cooperation of myopic students was higher than that of other intervention methods, and the feasibility of parental monitoring was also better.

Despite a multicenter randomized controlled trial suggesting that when RLRL treatment compliance is close to 75%, the effective rates for controlling axial length growth and SER progression could be as high as 76.8% and 87.7%, respectively [34]. Nevertheless, RLRL intervention has potential side effects, including transient or brief glare, flashes, and afterimages [128]. A two-year follow-up study of a randomized controlled trial cohort of participants who had used the RLRL device in the previous year for myopia control found that discontinuation of RLRL therapy in the second year significantly accelerated axial and refractive progression, not only compared to the first year (both *p* < 0.001) but also faster than the control and continuing treatment groups. This was accompanied by structural changes in choroidal thinning, suggesting a quantifiable rebound effect. Data from this follow-up study showed that the rate of AL progression in the discontinuation group was approximately 1.5 times that of the SVS control group and approximately 3.5 times that of the continuing RLRL group (multiple comparisons *p* = 0.002/<0.001). The rate of myopia progression in the SER group was approximately 1.7 times that of the control group and 4.6 times that of the continuing RLRL group (*p* = 0.003/<0.001). Comparisons before and after discontinuation revealed a significant decrease in choroidal thickness (ChT) from +5.47 μm to −18.23 μm in the discontinuation group compared to the first year (*p* = 0.002). In the second year, the ChT in the continuous RLRL group remained thicker, at +12.34 ± 18.78 μm. It is speculated that RLRL may maintain control of axial length growth by affecting choroidal vasodilation or blood supply, with some degree of rebound occurring upon discontinuation. Overall, the discontinuation group still outperformed the pure SVS group over the two years, indicating that the control effect achieved in the first year was not completely lost, although it was significantly reduced compared to the continuous RLRL group. However, it is worth noting that the second-year follow-up grouping in this study was voluntary rather than randomized, which poses a risk of selection bias and may have affected the current results to some extent. Future experimental research designs could optimize this issue [35]. Although most past studies did not report serious complications, one case report of transient macular damage aroused concern [130]. In general, published studies have focused on the effects of the RLRL therapeutic device on vision and safety, with insufficient attention paid to visual behavioral outcomes such as visual quality and binocular visual function. More importantly, its mechanism of action on the progression of myopia remains at the speculative level, lacking a complete mechanism chain and causal evidence that spans animal and human studies and covers from molecular to functional endpoints.

The RLRL therapeutic device is effective in controlling myopia; however, it is worth noting that the biphasic dose response is an important feature of PBMT [88]. RLRL therapy may also have such characteristics in the intervention of myopia prevention and control; that is, the light dose may have an inverted U-shaped relationship with efficacy. Low doses were ineffective, moderate doses were effective, and excessive doses weakened the effect or even caused damage. Due to the potential U-shaped effect of RLRL treatment, it is imperative to conduct mechanism-driven validation in the future. We need to administer the correct dosage, select the suitable endpoint, and ensure sufficient follow-up duration.

## Data Availability

No new data were created or analyzed in this study. Data sharing is not applicable to this article.

## Abbreviations

ADP, adenosine diphosphate; AL, axial length; ATP, adenosine triphosphate; Akt, Protein Kinase B; BNC, binuclear center; ${Ca}^{2+}$, calcium ions; cGMP, cyclic guanosine monophosphate; COL1A1, collagen type I alpha 1 chain; Cyt C, cytochrome c; ChT, choroidal thickness; DFCL, dual-focus design soft contact lenses; DIMS, defocus incorporated multiple segments; DOT, diffusion optics technology; $e^{-}$, electron; ECM, extracellular matrix; eNOS, endothelial nitric oxide synthase; ETC, electron transport chain; ERG, electroretinographic; FR, far-red; FMN, flavin mononucleotide; ${FMNH}_{2}$, reduced flavin mononucleotide; GJD2, gap junction protein delta 2; GWAS, large-scale genome-wide association studies; $H^{+}$, proton; HALT, highly aspherical lenslet target; HIF-1α, hypoxia-inducible factor 1-alpha; LEDs, light-emitting diodes; mRNA, messenger ribonucleic acid; MMP-2, matrix metalloproteinase-2; MAPK, mitogen-activated protein kinase; NIR, near-infrared; NMPA, national medical products administration; nNOS, neuronal nitric oxide synthase; NO, nitric oxide; $O_{2}$, oxygen; OK lens, orthokeratology lenses; PALs, progressive addition lenses; PBM, photobiomodulation; PBMT, photobiomodulation treatment; PI3K, phosphatidylinositol 3-kinase;PKG, cGMP-dependent protein kinase; PMF, proton motive force; Q, ubiquinone; $Q^{•-}$, semiquinone; $Q_{o}$, ubiquinone oxidation; Q cycle, quinone cycle; RLRL, repeated low-level red light; RASGRF1, ras protein specific guanine nucleotide releasing factor 1; ROS, reactive oxygen species; RET, reverse electron transport; RPE, retinal pigment epithelial; SER, spherical equivalent refraction; sGC, soluble guanylate cyclase; Smad, small mothers against decapentaplegic; SVLs, single-vision lenses; SFChT, sub-macular choroidal thickness; TCA, total choroidal area; TGA, therapeutic goods administration; TGF-β, transforming growth factor-beta; TRPV4, transient receptor potential vanilloid 4; VEGF, vascular endothelial growth factor; α-SMA, α-smooth muscle actin; ΔG, gibbs free energy change; ΔpH, pH gradient; ΔΨm, mitochondrial membrane potential; I, complex I of electron transport chain; II, complex II of electron transport chain; III, complex III of electron transport chain; IV (CCO), complex IV of electron transport chain (cytochrome c oxidase); V (ATP synthase), complex V of electron transport chain (adenosine triphosphate synthase); $O_{2^{•-}}$, superoxide anions.
